# The Role of the Methyltransferase Domain of Bifunctional Restriction Enzyme RM.BpuSI in Cleavage Activity

**DOI:** 10.1371/journal.pone.0080967

**Published:** 2013-11-04

**Authors:** Arthur Sarrade-Loucheur, Shuang-yong Xu, Siu-Hong Chan

**Affiliations:** New England Biolabs, Inc., Ipswich, Massachusetts, United States of America; Universität Stuttgart, Germany

## Abstract

Restriction enzyme (REase) RM.BpuSI can be described as a Type IIS/C/G REase for its cleavage site outside of the recognition sequence (Type IIS), bifunctional polypeptide possessing both methyltransferase (MTase) and endonuclease activities (Type IIC) and endonuclease activity stimulated by S-adenosyl-L-methionine (SAM) (Type IIG). The stimulatory effect of SAM on cleavage activity presents a major paradox: a co-factor of the MTase activity that renders the substrate unsusceptible to cleavage enhances the cleavage activity. Here we show that the RM.BpuSI MTase activity modifies both cleavage substrate and product only when they are unmethylated. The MTase activity is, however, much lower than that of M1.BpuSI and is thought not to be the major MTase for host DNA protection. SAM and sinefungin (SIN) increase the V_max_ of the RM.BpuSI cleavage activity with a proportional change in K_m_, suggesting the presence of an energetically more favorable pathway is taken. We further showed that RM.BpuSI undergoes substantial conformational changes in the presence of Ca^2+^, SIN, cleavage substrate and/or product. Distinct conformers are inferred as the pre-cleavage/cleavage state (in the presence of Ca^2+^, substrate or both) and MTase state (in the presence of SIN and substrate, SIN and product or product alone). Interestingly, RM.BpuSI adopts a unique conformation when only SIN is present. This SIN-bound state is inferred as a branch point for cleavage and MTase activity and an intermediate to an energetically favorable pathway for cleavage, probably through increasing the binding affinity of the substrate to the enzyme under cleavage conditions. Mutation of a SAM-binding residue resulted in altered conformational changes in the presence of substrate or Ca^2+^ and eliminated cleavage activity. The present study underscores the role of the MTase domain as facilitator of efficient cleavage activity for RM.BpuSI.

## Introduction

Restriction endonucleases (REases) have been central to cloning and mapping of DNA sequences [1]. In nature, REases are normally accompanied by methyltransferases (MTases) that recognize the same target sequences and modify specific adenines or cytosines by methylation so as to protect the host DNA from cleavage by the cognate REase. Restriction-modification (R-M) systems can be classified into four main types. Type I R-M systems are large protein complexes consisting of three unique subunits each contributing to a functional property of the enzymes: an M subunit (HsdM) that contributes to the methylation activity, an S subunit (HsdS) that contributes to the sequence specificity and an R subunit (HsdR) that contributes to the DNA cleavage activity. In methylation mode, Type I enzymes function as trimers in the form of M_2_/S_1_ whereas in cleavage mode they function as pentamers in the form of R_2_/M_2_/S_1_. Type II R-M systems normally contain separate MTases and REases which cleave at defined sites [2]. Type III R-M systems consist of Res and Mod subunits that associate to form a complex in order for DNA cleavage to occur, although the Mod subunits modify target bases on their own [3-5]. Type IV R-M systems encode proteins that recognize and cleave methylated cytosine-containing DNA [6,7] 

Research on Type II R-M systems has led to designations of subtypes that are descriptive of the properties of the REases. Type IIP REases, such as BamHI (G↓GATCC), recognize palindromic sequences and create symmetric double strand breaks, and are the most commonly used REases for molecular cloning. Type IIM REases recognize and cleave methylated, hydroxymethylated or glucosyl hydroxymethylated DNA [8-10]. Type IIS REases, such as BsaI (GGTCTC 1/5), cleave outside of the recognition site and are used in unconventional cloning methodologies such as Golden Gate Assembly [11]. This binary classification does not always capture all of the properties of the REases. For example, RM.BpuSI recognizes GGGAC and cleaves 10 and 14 nt downstream on the top strand and bottom strand, respectively (GGGAC 10/14). It contains both MTase and REase activity in one polypeptide and its cleavage activity can be enhanced by S-adenosyl-methionine (SAM). It can therefore be described as Type IIS/C/G for its cleavage site outside of the recognition sequence (Type IIS), bifunctional restriction/modification polypeptide (Type IIC), cleavage activity affected by S-adenosyl-methionine (SAM) (Type IIG). 

Several Type IIS/C/G REases enzymes have been reported, namely, GsuI [12,13], Eco57I ([12,13]), BseMII [14], Tth111II [15], MmeI [16,17] TaqII [18], TspDTI, TthHB27I, TsoI and TspGWI [15]. The stimulatory effect of SAM on cleavage activity has been well documented and presents a major paradox: a cofactor of the MTase activity that can modify the substrate such that it is no longer susceptible for cleavage enhances the cleavage activity. To date, the biochemical basis of the stimulatory effect remains largely unknown. The cloning of the BpuSI R-M system and the crystal structure of RM.BpuSI, the first of a Type IIS/C/G REase has recently been reported [19]. The BpuSI R-M system consists of three enzymes: two MTases and a dual function RM.BpuSI. The RM.BpuSI structure shows distinct functional domains arranged in the order of REase domain, a helix-rich connector domain, the MTase domain and target recognition domain (TRD) from the N- to C-terminus. The structure adopts an “idle” conformation where the REase active site is buried at the interface with the MTase domain and the TRD is expected to rotate relative to the MTase domain to assume the closed conformation adopted by M.TaqI as observed in co-crystal structures with substrate DNA [20]. This suggests that large movements of the REase domain and the TRD are involved for RM.BpuSI to carry out its catalytic activities. Here we report the biochemical properties of the RM.BpuSI MTase activity and provide evidence to suggest that SAM enhances cleavage activity by facilitating domain movements.

## Materials and Methods

### Protein expression and purification

**Figure 1:**
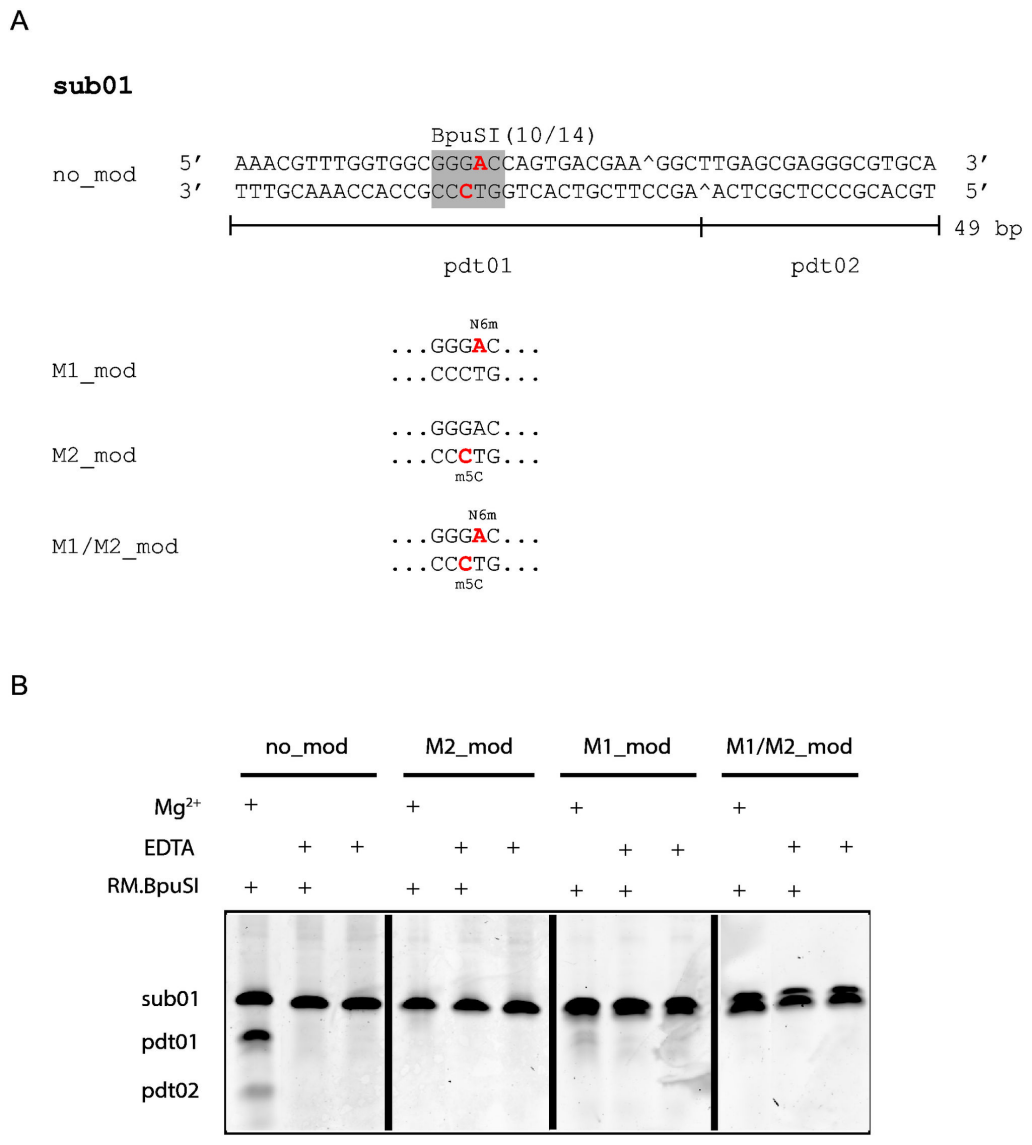
Oligonucleotide duplexes used in this study and their susceptibility to RM.BpuSI cleavage. (A) Sub01 is a 49-bp synthetic oligonucleotide duplex containing a single BpuSI site. Site-specific modified versions of sub01 were also synthesized. M1-modified sub01 (M1_mod) contains a N6mA (red) on the top strand. M2-modified sub01 (M2_mod) contains a m5C (red) on the bottom strand. Oligonucleotide duplex M1/M2_mod contains both modified bases. Pdt01 and pdt02 are the RM.BpuSI cleavage products. (B) RM.BpuSI REase activity. RM.BpuSI cleaved unmodified sub01 into expected fragments. Substrates containing M2- or M1/M2-modifications were not cleaved. A small amount of cleavage product is observed with M1-modified substrate.

The *bpuSIRM* gene, which encodes for the full-length RM.BpuSI gene containing the REase and MTase domains and target recognition domain (TRD), was PCR-amplified and cloned into pT7SL (a pTXB1 (NEB) derivative without the *Mxe GyrA* intein ORF). Chemically competent *E. coli* cells ER3081 (*fhuA2 lacZ::T7 gene1[lon*]* ompT gal attB::pCD13-lysY, lacIq sulA11 R(mcr-73::miniTn10--TetS*)*2  [dcm*]* R(zgb-210::Tn10 --TetS*)* endA1 ∆(mcrC-mrr*)*114::IS10*) carrying pACYC-*bpuSIM1M2* was transformed by pT7SL-*bpuSI* and plated out on LB plates containing 100 μg/ml ampicillin and 30 μg/ml chloramphenicol. Expression was done as described before [19]. The supernatant of lysed cells was loaded onto a HiTrap Heparin column (5 ml; GE Life Sciences) in a buffer containing 20 mM Tris HCl, pH 8.0, 100 mM NaCl. Peak fractions eluted from a linear NaCl gradient were pooled, diluted in a buffer containing 20 mM potassium phosphate, pH 7.0, 50 mM NaCl and loaded onto a ceramic hydroxyapatite column (7 ml; Bio-Rad). Peak fractions eluted from a linear potassium phosphate gradient were pooled and concentrated to ~1.5 ml (4 mg/ml) using a Centricon 70 plus concentrator (MWCO = 10 kDa; Millipore). To remove SAM potentially bound to and co-purified with RM.BpuSI, 5 mg (50 nmol) of the concentrated protein were incubated with 100 nmol of a oligonucleotide duplex containing a BpuSI site (sub01, Figure 1) in a 1-ml solution containing 20 mM Tris HCl, pH 8.0, 50 mM NaCl, 5 mM DTT, 1 mM EDTA at 37°C for 1 h. The reaction was then diluted in a buffer containing 20 mM Tris HCl, pH 8.0, 50 mM NaCl and loaded into a Source 15Q column (7 ml; GE Life Sciences). Peak fractions eluted from a linear NaCl gradient were pooled, concentrated and stored at -20°C in a buffer containing 25 mM Tris, pH 7.5, 300 mM NaCl, 40% glycerol. The purity of the protein preparations was evaluated using SDS-PAGE followed by Coomassie Blue staining (SimplyBlue SafeStain; Invitrogen). The *bpuSIM1* ORF was PCR-amplified and cloned into pT7SL. The construct pT7SL-*bpuSIM1* was used to transform ER3081. Expression and purification procedures were essentially the same as for RM.BpuSI. 

### Methyltransferase activity assays

To determine the target base specificity of M1.BpuSI, M1.BpuSI was produced by using PURExpress *in vitro* transcription/translation system (NEB) in a 25 μl reaction according to manufacturer’s instructions. 0.6 pmol of a 2 kb fragment derived from pSYX20 (NEB) containing an HgaI site overlapping with a BpuSI site (GGGACGC; HgaI site being GACGC) was incubated with 2 μl of the PURExpress product in 20 μl reactions containing 20 mM Tris HCl, pH 8.0, 50 mM NaCl and 160 μM SAM at 37°C for 2 h. 2 μl of 10x NEBuffer 1 and 2 U of HgaI (NEB) were added to the reaction and incubation at 37°C was continued for 1 h. The cleavage reactions were then analyzed by electrophoresis through a 0.8% agarose gel. HgaI cleavage activity is blocked by methylation at the N6 position of the lone adenine of GACGC (Stickel, S.K. & Roberts, R.J., REBASE). The absence of HgaI cleavage on the BpuSI/HgaI overlapping site is indicative of methylation activity of N6A methylation activity on GGGAC by M1.BpuSI.

For endpoint MTase activity assays, 1 pmol of annealed oligonucleotide duplexes was modified by 8 pmol of purified M1.BpuSI or RM.BpuSI in 100 μl reactions containing 20 mM Tris HCl, pH 8.0, 100 mM NaCl, 0.5 μM of ^3^H-SAM (Perkin-Elmer) and 5 mM EDTA or MgCl_2_ at 37°C for 1 h in triplicate. The reactions were stopped by immersing in a dry ice-ethanol bath and stored at -20°C until analyzed. It has been determined separately that the amount of modified sub01 DNA plateaued after 30 minutes of incubation with molar excess of RM.BpuSI (data not shown). Ten microliters of the stopped reactions were spotted onto Whatman DE81 discs (2.4 cm; GE Healthcare) in triplicate. The discs were air-dried and washed three times by 0.2 M ammonium bicarbonate, water and then ethanol. After air-drying, radioactivity remained on the discs was detected by scintillation counting. 

### Endonuclease activity assays

RM.BpuSI endonuclease activity was routinely assayed by incubating 5 μl of 2-fold serially diluted enzyme in 50 μl reactions containing 1x NEBuffer 4, 1 μg λ DNA, with or without 160 μM SAM at 37°C for 1 h. The reactions were stopped by adding 10 μl of 6x DNA loading dye (NEB) and analyzed by electrophoresis through 1% agarose in TBE.

Forty-nine bp long oligonucleotide duplexes containing a unique BpuSI site (sub01) were synthesized by IDT (Coralville, Iowa) with or without the indicated ^N6m^A on the top strand or ^m5^C on the bottom strand. The oligonucleotide duplexes were annealed and incubated with 0.2 μM of RM.BpuSI in 20 mM Tris HCl, pH 8.0, 100 mM NaCl, 5 mM MgCl_2_, at 37°C for 1 h. The reactions were stopped by adding a 6x gel loading dye (NEB) and analyzed by electrophoresis using 10% polyacrylamide gels (Invitrogen) followed by SYBR Gold staining (Invitrogen). The stained gels were scanned and analyzed using Typhoon 9400 and the accompanying ImageQuant TL software (GE Healthcare).

Steady state kinetics experiments were carried out by incubating 200 nM of purified RM.BpuSI protein with 1.25 to 25 μM of sub01with a 5’ FAM label [21] on the top strand in the presence or absence of 160 μM of SAM or sinefungin (SIN) in 100 μl reactions containing 20 mM Tris HCl, pH 8.0, 100 mM NaCl, 5 mM MgCl_2_ at 37°C for 1 h. Five microliter samples were removed at designated time points and analyzed on a 10% TBE polyacrylamide gel. The gels were analyzed using Typhoon 9400 and ImageQuant TL. Protein concentration was determined by measuring the OD at 280 nm and using an extinction coefficient of 100000 M^-1^cm^-1^ and a molecular weight of 99.5 kDa.

### Spectrophotometric analysis of RM.BpuSI-ligand interactions

8-anilino-1-naphthalene sulfonic acid (ANS; Sigma-Aldrich) interacts with proteins with dissociation constant in the sub-millimolar range [22]. It interacts with amino acids through electrostatic and hydrophobic interactions [22] and has been used as a probe to detect conformational changes of RM.MmeI upon interactions with SAM [23]. Purified RM.BpuSI WT or mutant N406A (2.5 μM) which had undergone the SAM-removal treatment was incubated with 400 μM ANS in a buffer containing 20 mM Tris HCl, pH 8.0, 100 mM NaCl at 25°C for 5 min before ligands were added. Ligands include SAM (160 μM), SIN (160 μM), CaCl_2_ (2 mM), sub01 (80 μM) and pdt01 (80 μM). Fluorescence spectra were collected from 450 to 600 nm with excitation at 360 nm. The binding reactions were assembled with utmost attention to variability and each data point represents the average of 6 scans. The change in fluorescence ΔF was calculated as ΔF = F_enz_ - F_no_enz_. For the calculation of equilibrium dissociation constant, the ligands were titrated into 100 μl of 2.5 μM of RM.BpuSI in the same buffer. After subtracting from the zero ligand value, the ΔF values at 490 nM were plotted against ligand concentrations. The data points were fitted to a hyperbolic function: ΔF = (ΔF_max_ * [L])/(K_d_-[L]) where ΔF_max_ is the maximum change in fluorescence, [L] is ligand concentration and K_d_ is the dissociation constant. 

## Results

### Target bases of M1.BpuSI and M2.BpuSI

RM.BpuSI is a fusion of MTase and REase [19]. It recognizes a 5-bp sequence GGGAC and cleaves 10 nt and 14 nt downstream at the top strand and the bottoms strand, respectively (GGGAC 10/14). Curiously, the BpuSI R-M system contains two more MTases: M1.BpuSI, M2.BpuSI [19].. Amino acid sequence analysis suggested that both M1.BpuSI and RM.BpuSI contain the N6A MTase active site motif. The RM.BpuSI crystal structure shows that the MTase domain is structurally similar to M.TaqI (TCG^m6^A). To determine the target base for M1.BpuSI, a substrate DNA containing overlapping BpuSI and HgaI sites (GGGACGC; HgaI site being GACGC) modified *in vitro* by M1.BpuSI was subjected to cleavage by HgaI. HgaI cleavage is blocked by N6 methylation of the lone adenine of the HgaI site (Stickel, S.K. & Roberts, R.J., REBASE). Our results showed that the modified DNA was resistant to cleavage by HgaI (data not shown), suggesting that M1.BpuSI modifies the lone adenine base on the top strand. 

The target base of M2.BpuSI was determined to be the first cytosine of the bottom strand (GTCCC) through bisulfite sequencing of a BpuSI site on a plasmid carrying the M2.BpuSI ORF under the control of a T7 promoter (data not shown). These results were consistent with the target base specificity of the two MTases of the homologous BspLU11III system [24,25]. 

To verify the functional role of the modifications, synthetic oligonucleotide duplexes containing the M1- and/or M2-modifications (Figure 1A) were subjected to RM.BpuSI cleavage. Figure 1B shows that the M2- and M1/M2-modification completely blocked RM.BpuSI cleavage *in vitro*. A very small amount of cleavage product can be observed wit the M1-modified sub01.

Although M2.BpuSI modification alone blocks RM.BpuSI cleavage *in vitro* (Figure 1B), omission of M1.BpuSI expression in *E. coli* lead to unstable RM.BpuSI clones (data not shown), indicating that both M1 and M2.BpuSI are required for the establishment of a stable RM.BpuSI system *in vivo*. It is in contrast to RM.MmeI, another well-studied Type IIS/C/G REase and its homologous REases which only require modification on one DNA strand for host protection and do not have a separate companion MTase [17]. 

### Both cleavage substrate and cleaved product are substrates for RM.BpuSI MTase activity

**Figure 2:**
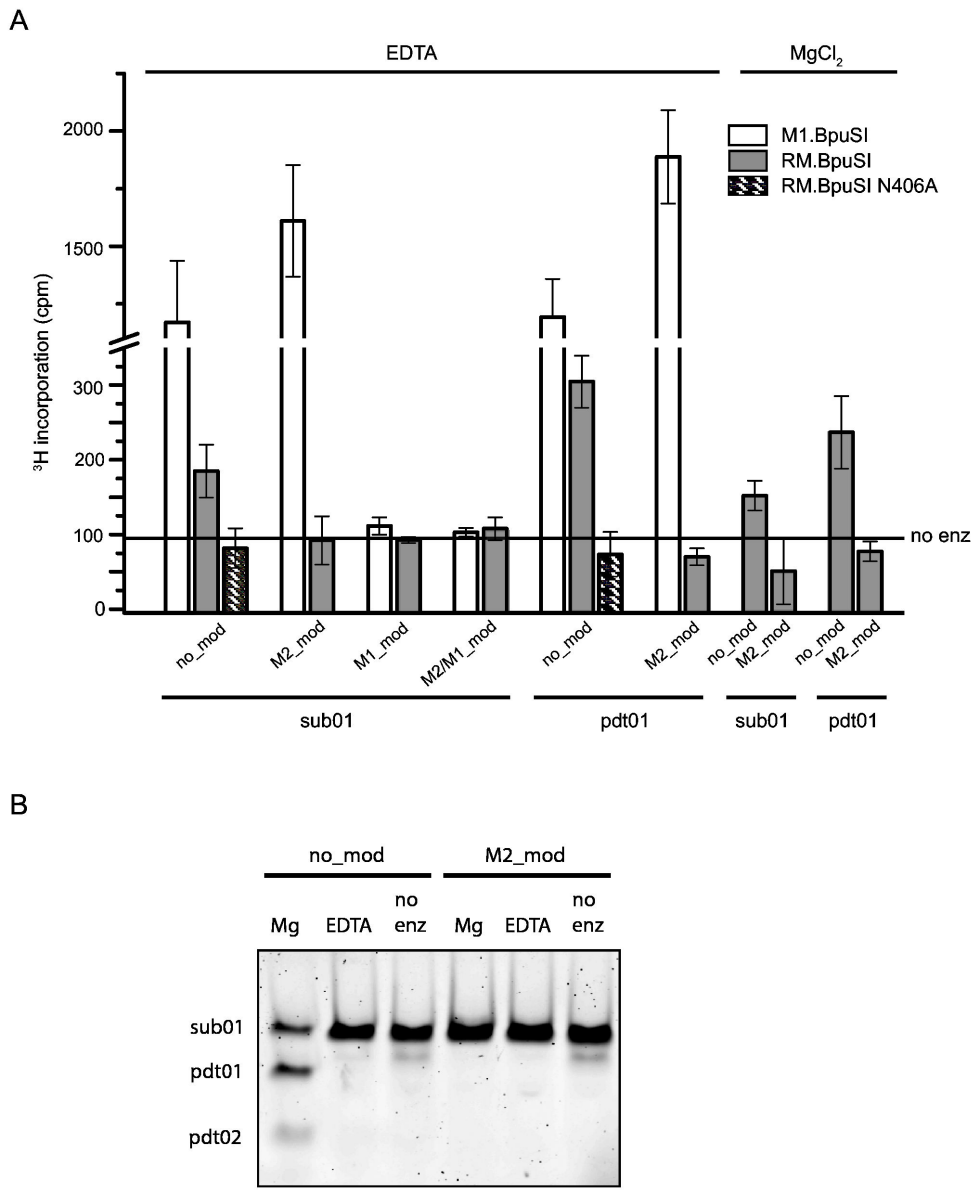
Substrate specificity of RM.BpuSI MTase activity. (A) *In* *vitro* MTase activity. Indicated oligonucleotide duplexes were incubated with 8-fold molar excess of purified WT or N406A RM.BpuSI or M1.BpuSI in the presence of 0.5 μM ^3^H-SAM and 5 mM EDTA or MgCl_2_ at 37°C for 1 h. Radioactivity incorporation was measured by scintillation counting filter-bound DNA.. Values shown are average of triplicate experiments and subtracted from background readings without enzyme added. Error bars represent standard errors from the triplicates. (B) Unmodified but not M2-modified sub01 were cleaved into expected products in the presence of Mg^2+^ in the MTase activity assays.

To understand the role of the MTase domain in RM.BpuSI, we first investigated the substrate preference of the M1.BpuSI and RM.BpuSI MTase. Because RM.BpuSI cleaves downstream of the recognition sequence and does not destroy the recognition site, methylation is possible before and after RM.BpuSI cleavage. *In vitro* endpoint MTase activity assays were performed using oligonucleotide duplexes as cleavage substrate and cleavage product with or without the M1 or M2 modification (Figure 1A). Excess enzyme was used over substrate so that substrate binding and catalysis are considered simultaneously, assuming the rate of product release is the same for all substrates. The reactions were carried out in the presence of 5 mM EDTA to inhibit the RM.BpuSI endonuclease activity. Endpoint experiments showed that both RM.BpuSI and M1.BpuSI methylated both the unmodified cleavage substrate and the unmodified cleavage product (Figure 2A). However, the MTase activity of RM.BpuSI is 4-6 fold lower than that of M1.BpuSI. While M1.BpuSI modified M2-modified substrate, RM.BpuSI did not modify this substrate to any observable level. Moreover, M1.BpuSI modified the cleavage substrate and cleavage product to a similar level. RM.BpuSI appeared to modify the cleavage product to a slightly higher level than the cleavage substrate, but kinetic experiments performed in the presence of excess enzyme over substrate did not show significant difference of specific MTase activity between the two substrates (data not shown). Compared to M1.BpuSI, the relatively low MTase activity towards unmodified substrates and the lack of activity toward M2-modified substrates suggests that the RM.BpuSI MTase activity is not the major MTase for the protection of the host BpuSI sites from cleavage *in vivo*. 

Next we asked if RM.BpuSI performs both cleavage and modification reactions if both SAM and Mg^2+^ are present. Our results showed that in the presence of MgCl_2_, RM.BpuSI modified the DNA to a similar extent as in the presence of EDTA (Figure 2A). Analysis of the MTase assay reactions by 10% polyacrylamide gel electrophoresis showed that sub01 was cleaved into the expected products when 5 mM of MgCl_2_ was present (Figure 2B). In the presence of SAM and Mg^2+^, it is possible that both cleavage and methylation reactions take place in parallel. Our conformational studies showed that in the presence of substrate, SIN and Ca^2+^, RM.BpuSI adopts a cleavage but not MTase conformation (see below), suggesting the enzyme exhibits MTase activity in the cleavage conformation.

### SAM and SIN enhance the cleavage reaction by facilitating conformational changes

**Figure 3:**
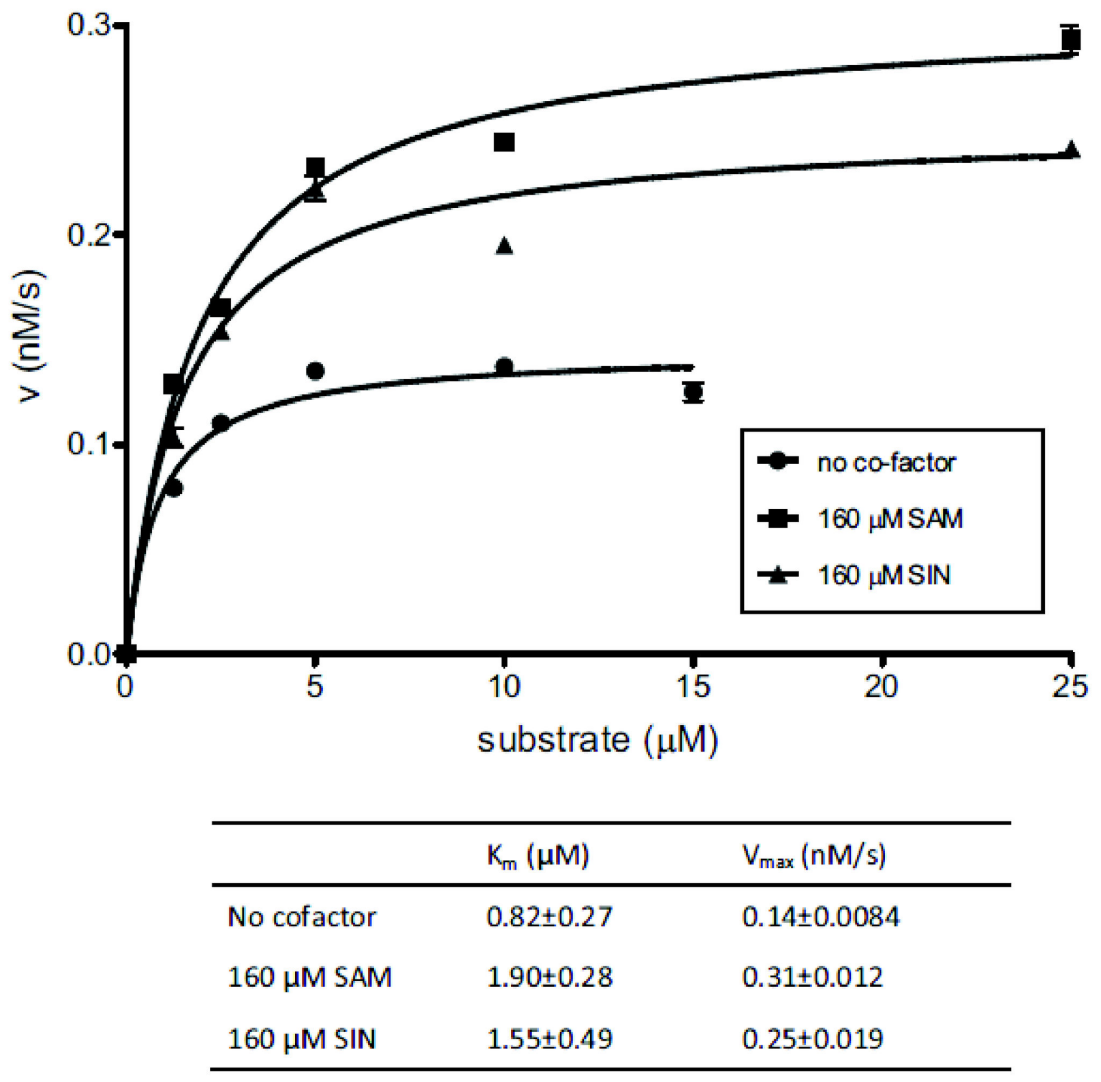
Effect of SAM and SIN on the steady state kinetics of cleavage activity. Steady state kinetics experiments of RM.BpuSI cleavage activity were carried out by incubating 200 nM of purified RM.BpuSI protein with 1.25 to 25 μM of FAM-labeled sub01 at 37°C for 1 h in the presence or absence of 160 μM of SAM or SIN. The initial rate (v) for each substrate concentration was plotted against substrate concentration. K_m_ and V_max_ values were obtained by fitting the data points to the Michaelis-Menten equation. Error bars indicate the standard deviation of the fitting of the initial linear rate of each data point.

Many Type IIG REases exhibit higher cleavage activity in the presence of cofactor SAM [15,26] but not much is known about how it is achieved. We tried to answer this question by investigating how SAM affects the REase activity of RM.BpuSI. Cleavage reactions were carried out by incubating FAM-labeled sub01 with 200 nM purified RM.BpuSI that had undergone the SAM-removal treatment under steady state conditions (see Material and Methods). A plot of the rate of product formation against substrate concentration and the derived kinetic parameters are shown in Figure 3. In the presence of SAM, the V_max_ of the RM.BpuSI REase reaction increased by ~2 fold with the K_m_ value increased by the same magnitude compared to the kinetics in the absence of SAM (Figure 3). The enhancement of V_max_ is independent of the MTase activity because SIN, a non-permissive analog of SAM, caused a similar increase in the V_max_ and K_m_ values. The concerted increase in V_max_ and K_m_ suggests that the enzyme carries out the cleavage reaction through a more favorable energy landscape when SAM or SIN is present. 

**Figure 4:**
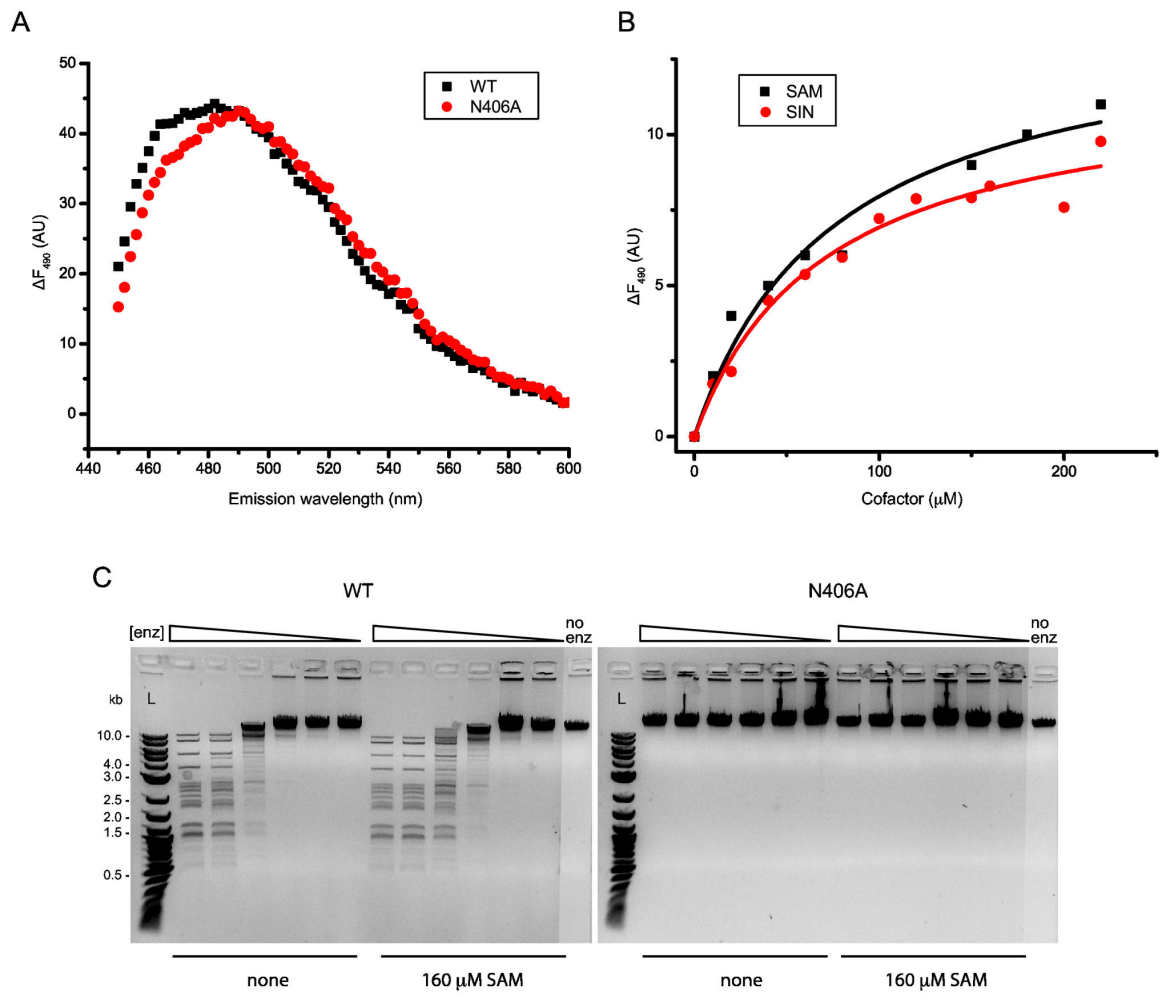
Mutation of N406 in the NPPY motif abolishes MTase and cleavage activity. (A) Fluorescence spectra of WT and N406A RM.BpuSI. The high similarity of the two spectra indicated that mutation N406A does not induce significant conformation change to RM.BpuSI. Titration of SAM or SIN into mutant N406A does not show specific changes in fluorescence (data not shown). (B) Titration of SAM or SIN into WT RM.BpuSI in the presence of 400 μM ANS. The data points are fitted to a hyperbolic function and K_d_ values are found to be 76.4 and 71.1 μM for SAM and SIN, respectively. (C) Two-fold dilutions of WT or N406A RM.BpuSI was incubated with 1 μg of λ DNA in the presence or absence of 160 μM SAM at 37°C for 1 h. While WT exhibits enhanced cleavage activity in the presence of SAM, mutant N406A does not exhibit cleavage activity in the presence or absence of SAM.

To investigate the role of SAM-binding to the cleavage activity of RM.BpuSI, the SAM-binding residue N406 within the NPPY motif was mutated to alanine. Spectroscopic analysis of protein conformation using ANS as a probe showed that mutant N406A adopted a conformation highly similar to that of WT RM.BpuSI (Figure 4A). Titration of SAM or SIN showed that the WT RM.BpuSI binds to SAM and SIN at a K_d_ of 76.4±18.2 and 71.1±14.2 μM, respectively (Figure 4B). Mutant N406A did not bind to SAM or SIN specifically (data not shown) and was inactive in MTase as expected (Figure 2A). To our surprise, this SAM-binding deficient mutant is also inactive in cleavage (Figure 4C). Two possibilities can reconcile the presence of cleavage activity after SAM-removal treatment of WT RM.BpuSI observed in Figure 4C and elsewhere (data not shown): 1. Our SAM-removal treatment is not complete. The presence of cleavage activity is due to the presence of a small amount of SAM-bound RM.BpuSI molecules; 2. The mutant N406A adopts conformations that are not competent of cleavage upon binding to divalent metal ion and/or substrate. 

### RM.BpuSI undergoes conformational changes in the presence of activity-related ligands

**Figure 5:**
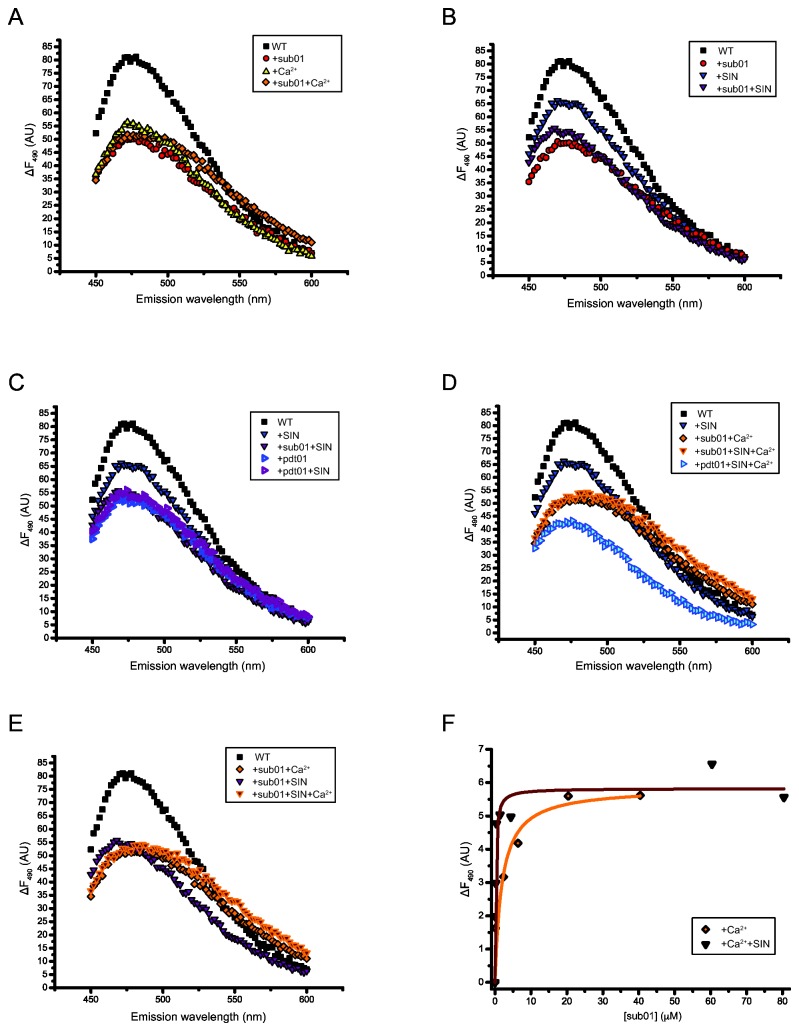
Fluorescence spectra of RM.BpuSI-bound ANS as surrogate of protein conformations. (A) Pre-cleavage and cleavage states. Fluorescence spectra of RM.BpuSI alone, with Ca^2+^, sub01 and sub01+Ca^2+^ are shown. The Ca^2+^, sub01 and sub01+Ca^2+^ spectra have significant lower maximum emission intensity from that of the WT RM.BpuSI alone, suggesting substantial conformational changes upon binding to the ligands. The Ca^2+^, sub01 and sub01+Ca^2+^ spectra resembled each other in shape and emission maximum and are inferred as representing the pre-cleavage/cleavage state. (B) and (C) SIN induces the MTase conformation. Fluorescence spectra of RM.BpuSI alone, with sub01, SIN and SIN and sub01 are shown. In the presence of SIN, RM.BpuSI adopts a conformation different from the SIN or Ca^2+^-bound conformation. The RM.BpuSI+sub01+SIN spectrum exhibits a higher maximum emission intensity than the RM.BpuSI+sub01 spectrum (B). The fluorescence spectra of RM.BpuSI alone, with SIN, pdt01, SIN+sub01 or SIN+pdt01 are shown (C). The spectra of sub01+SIN and pdt01+SIN are indistinguishable, suggesting an MTase state conformation. The spectrum of pdt01 is also indistinguishable from the SIN-bound spectra, suggesting that binding of pdt01 in the absence of divalent metal ions induces the MTase state. (D) The RM.BpuSI+sub01+Ca^2+^+SIN spectrum is highly similar to that without SIN, especially in the lower emission wavelengths, suggesting that the presence of SIN does not induce significant conformational change to the RM.BpuSI-M^2+^-substrate complex. The fluorescence spectrum of RM.BpuSI+pdt01+Ca^2+^+SIN is significantly different from any of the spectra, suggesting that the enzyme undergoes more conformational changes after the cleavage reaction and remains bound to the product. (E) In the presence of sub01, SIN and Ca^2+^, the fluorescence spectrum of RM.BpuSI-bound ANS resembles the RM.BpuSI+sub01+SIN+Ca^2+^ (cleavage conformation) spectrum but not the RM.BpuSI+sub01+SIN spectrum (MTase conformation). (F) Sub01 was titrated into a solution containing 2.5 μM RM.BpuSI and 2 mM CaCl_2_ in the presence or absence of 160 μM SIN. Fluorescence change (ΔF) relative to zero sub01 conditions was plotted against the final concentrations of sub01. The dissociation constant K_d_ is 0.17 or 1.9 μM in the presence or absence of SIN, respectively.

We further investigated the conformational changes of WT RM.BpuSI and mutant N406A by measuring the fluorescence spectrum of bound ANS in the presence of ligands. SIN and Ca^2+^ are used instead of SAM and Mg^2+^, respectively, because they are non-permissive co-factors for MTase and cleavage activity, respectively, and therefore allow us to measure the equilibrium state of the enzyme-ligand complexes. Figure 5A shows the fluorescence spectra of RM.BpuSI alone or in the presence of sub01, CaCl_2_ or both. The presence of either of the ligands resulted in substantial reduction of maximum emission intensity, suggesting that RM.BpuSI undergoes significant conformational changes upon binding to Ca^2+^ or sub01. The fluorescence spectra in the presence of sub01 and CaCl_2_ are highly similar in shape and emission maximum, suggesting pre-cleavage states of slightly different conformation. In the presence of both sub01 and Ca^2+^, the fluorescence spectrum undergoes a small red shift, suggesting another conformational change, possibly to a cleavage conformation. The difference, however, is rather small and we therefore interpret the results as an indication of a pre-cleavage/cleavage conformation distinct from the apo enzyme conformation. 

We next examined the effect of SIN in the conformation of RM.BpuSI in the presence of sub01. Figure 5B shows that in the presence of SIN, RM.BpuSI adopts a conformation drastically different from the sub01 or Ca^2+^-bound or apo conformation. This unique conformation is inferred as an important state to achieve the cleavage state (see below). The RM.BpuSI-sub01-SIN spectrum exhibits a higher fluorescence intensity than the RM.BpuSI-sub01. Further experiments using pdt01 as ligand showed that the RM.BpuSI-pdt01-SIN and RM.BpuSI-sub01-SIN spectra are indistinguishable (Figure 5C), suggesting that RM.BpuSI adopts the same conformation when sub01 or pdt01 was bound in the presence of SIN. Our biochemical experiments showed that both sub01 and pdt01 are substrate for the RM.BpuSI MTase activity in the presence of SAM and EDTA (absence of divalent metal ion). This sub01-SIN/pdt01-SIN conformation could therefore represent the MTase state. Interestingly, the RM.BpuSI-pdt01 spectrum is also indistinguishable from the RM.BpuSI-pdt01-SIN and RM.BpuSI-sub01-SIN spectra (Figure 5C), suggesting that in the absence of divalent metal ions, binding of the cleavage product transforms RM.BpuSI into the MTase state. 

We further examined the effect of SIN on the RM.BpuSI-sub01-Ca^2+^ cleavage complex. Figure 5D shows that the RM.BpuSI-sub01-Ca^2+^-SIN spectrum is highly similar to that without SIN, especially in the lower emission wavelengths. This indicates that SIN does not induce significant conformation changes in the RM.BpuSI-Ca^2+^-sub01 cleavage complex. Furthermore, the RM.BpuSI+pdt01+Ca^2+^+SIN spectrum is significantly different from any of the spectra, suggesting that the enzyme undergoes more conformational changes after the cleavage reaction and remains bound to the product (Figure 5D), suggesting that further conformational changes occur after the cleavage chemistry, and that the enzyme remains bound to the product. 

To find out if SAM-bound RM.BpuSI-substrate-divalent metal ion complex prefers cleavage or MTase activity, we compared the fluorescence spectra of RM.BpuSI in the presence of SIN, Ca^2+^ and sub01 to those represent the MTase or cleavage conformations. Figure 5E shows that in the presence of sub01, SIN and Ca^2+^, the fluorescence spectrum of the bound ANS resembles the RM.BpuSI+sub01+Ca^2+^ spectrum (the cleavage conformation) than to the RM.BpuSI+sub01+SIN spectrum (the MTase conformation). This suggests that the majority of the enzyme is engaged in the cleavage mode. The fact that RM.BpuSI exhibits similar level of MTase activity in the presence or absence of Mg^2+^ (Figure 2A) suggests that RM.BpuSI can carry out MTase activity in both MTase and cleavage conformation. 

As a control, the RM.BpuSI cleavage activity was assayed in the presence or absence of 0.4 mM ANS. We found that the presence of 0.4 mM ANS does not affect the RM.BpuSI cleavage activity under standard conditions on λ DNA (data not shown). 

### SIN increases the binding affinity of substrate to RM.BpuSI under cleavage conditions

To further investigate the role of SIN in the cleavage activity, equilibrium dissociation constant of sub01 to WT RM.BpuSI under cleavage conditions was determined in the presence or absence of SIN. As shown in Figure 5F, the K_d_ of sub01 binding to Ca^2+^-bound RM.BpuSI is 0.17 and 1.6 μM in the presence or absence of SIN, respectively. The presence of SIN therefore increases the binding affinity of the substrate to RM.BpuSI under cleavage conditions.

### SAM-binding deficient mutant N406A adopts cleavage-incompatible conformations in the presence of metal ions or substrate

**Figure 6:**
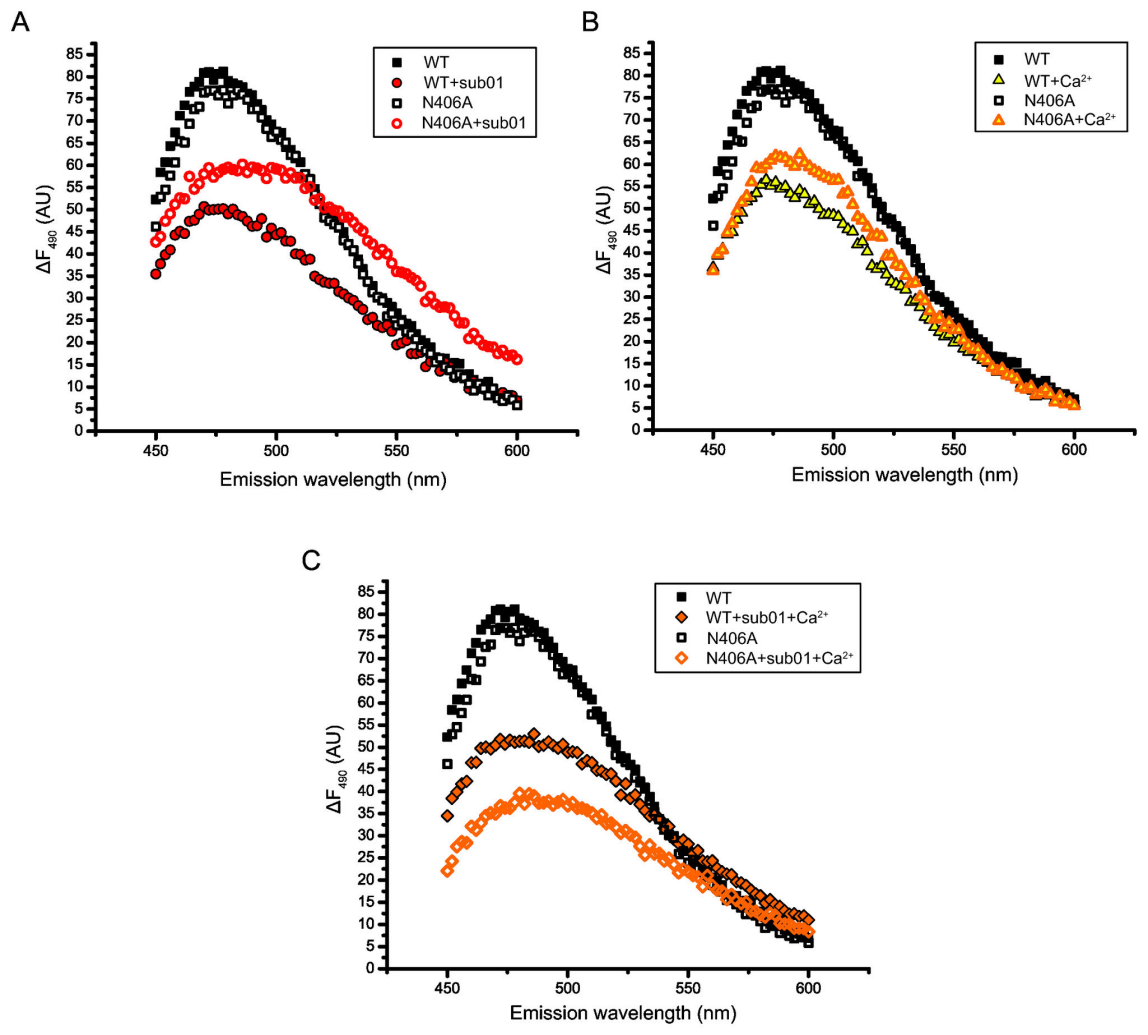
Mutation N406A results in conformations incompatible to cleavage upon binding with Ca^2+^ and/or substrate. The fluorescence spectra of RM.BpuSI WT or mutant N406A alone and with sub01 (A) or CaCl_2_ (B) or with sub01 and CaCl_2_ (C) are shown. Mutant N406A adopts conformations very different from that of the WT in the presence of the ligands.

To determine if the absence of cleavage activity of mutant N406A is due to alternative conformational changes upon substrate binding, fluorescence spectra of N406A were obtained in the presence and absence of sub01 or CaCl_2_. Figure 6 shows that mutant N406A adopts a very different conformation than the WT when sub01 (Figure 6A) or Ca^2+^ (Figure 6B) is present. Similarly, when both Ca^2+^ and sub01 are present, mutant N406A adopts yet another different conformation (Figure 6C), supporting the notion that mutant N406A adopts conformations that are not compatible to the cleavage activity upon substrate or Ca^2+^ binding.

## Discussion

SAM has been shown to affect the cleavage activity of Type I and Type III REases [27-29]. It induces conformational changes required for cleavage activity in EcoKI, a prototypical Type I REases [5,27], and acts as a positive allosteric effector for the cleavage activity of EcoP1I, a prototypical Type III REase [29]. Although SAM does not normally affect mono-functional Type II REases, cleavage activity of Type IIG REases is affected by SAM [15,26]. The mechanism through which SAM influences Type IIG REases cleavage activity, however, remains unclear. 

In the crystal structure, RM.BpuSI adopts an “idle” configuration: the cleavage active site is buried at the interface between the REase domain and the MTase domain. The TRD is in an “open” conformation with a S-adenosyl-homocysteine, the byproduct of MTase reaction, bound to the MTase active site [19]. Molecular modeling predicted that a backbone rotation at a hinge point located at or near T580 would bring the RM.BpuSI TRD into the same position of the M.TaqI TRD bound to the substrate DNA (PDB 2IBS). A further rotation at or near A170 and G171 would position the REase active site to cleave the substrate DNA at the cleavage site [19]. 

In this report, we showed that under steady state conditions, SAM and SIN increased the V_max_ of the RM.BpuSI cleavage activity with a proportional change in the K_m_ value. Without the knowledge of the oligomerization state during cleavage, we cannot calculate the k_cat_ or the k_cat_/K_m_ values from the steady state kinetics experiments. Nonetheless, if we assume that SAM and SIN do not alter the enzyme’s oligomerization state during cleavage, the V_max_ value and the V_max_/K_m_ ratio can been seen as a surrogate of the k_cat_ and the k_cat_/K_m_ value (specificity constant), respectively. In that case the V_max_/K_m_ value of RM.BpuSI cleavage activity in the absence or the presence of SAM or SIN would be 0.00017, 0.00016 and 0.00016 s^-1^, respectively. SAM and SIN therefore do not alter the V_max_/K_m_, and hence the k_cat_/K_m_ value. Therefore, SAM and SIN increase the rate of cleavage through a higher turnover rate and an energetically more favorable pathway. A higher turnover rate is also evident by comparing the equilibrium dissociation constant K_d_ and the K_m_ values from the steady state kinetics experiments. In the presence of SIN, the K_d_ value decreases from 1.6 μM to 0.17 μM; K_m_ increases from 0.82 μM to 1.55 μM. In contrast to the K_d_ value that represents the mass partitioning of substrate-bound enzyme versus apo enzyme under equilibrium conditions, K_m_ is a kinetic representation of the enzyme turnover rate divided by the effective rate of substrate binding (K_m_ = k_cat_/(k_cat_/K_m_)). Whereas a decrease in K_d_ value indicates that SIN increases the binding affinity of the substrate DNA to the enzyme, an increase of K_m_ at the same time is conceivable when the turnover rate increases at a larger extent than the increase in the rate of substrate binding.

Using bound ANS as a probe to protein conformation, we identified distinct functional conformations adopted by RM.BpuSI in the presence of relevant ligands: the pre-cleavage/cleavage state in the presence of substrate or Ca^2+^, substrate DNA or both; the MTase state in the presence of SIN and substrate, SIN and cleavage product or cleavage product alone. Interestingly, RM.BpuSI adopts a unique conformation when SIN is bound. This unique conformation is inferred as an intermediate of an energetically favorable pathway responsible for the increase in V_max_ in cleavage. Our results of increased enzyme turnover rate and binding affinity of sub01 to RM.BpuSI in the presence of SIN in the presence of Ca^2+^ (Figure 5F) support this hypothesis.

Finally, we created and purified a SAM-binding deficient mutant N406A to investigate if the absence of SAM-binding would obviate the increase the V_max_ for RM.BpuSI cleavage activity. To our surprise, this mutant is inactive in cleavage. After verifying that this mutant adopts the same conformation as the WT protein in the absence of ligands, we went on to show that this mutant adopts conformations different from the cleavage conformations of the WT when Ca^2+^ or sub01 is present (Figure 6). The results with this SAM-binding deficient mutant show that the SAM-binding domain of RM.BpuSI does not only increase the V_max_ of cleavage activity by providing a energetically favorable pathway but also making sure the correct conformational changes take place. It has been reported that SAM induces conformational changes in RM.MmeI and enhances the binding of the enzyme to target DNA [23]. The role of the MTase domain could therefore be a common regulatory mechanism for the cleavage activity of Type IIC/G REases.
